# In Silico and In Vitro Studies of *Alchemilla viridiflora* Rothm—Polyphenols’ Potential for Inhibition of SARS-CoV-2 Internalization

**DOI:** 10.3390/molecules27165174

**Published:** 2022-08-14

**Authors:** Relja Suručić, Jelena Radović Selgrad, Tatjana Kundaković-Vasović, Biljana Lazović, Maja Travar, Ljiljana Suručić, Ranko Škrbić

**Affiliations:** 1 Department of Pharmacognosy, Faculty of Medicine, University of Banja Luka, 78000 Banja Luka, Bosnia and Herzegovina; 2 Department of Pharmacognosy, Faculty of Pharmacy, University of Belgrade, VojvodeStepe 450, 11221 Belgrade, Serbia; 3 Internal Medicine Clinic, Division of Pulmonology, University Clinical Hospital Center Zemun, 11080 Belgrade, Serbia; 4 Department of Microbiology, Faculty of Medicine, University of Banja Luka, 78000 Banja Luka, Bosnia and Herzegovina; 5 Department of Organic Chemistry, Faculty of Medicine, University of Banja Luka, 78000 Banja Luka, Bosnia and Herzegovina; 6 Department of Pharmacology, Toxicology and Clinical Pharmacology, Faculty of Medicine, University of Banja Luka, 78000 Banja Luka, Bosnia and Herzegovina

**Keywords:** *Alchemilla viridiflora* Rothm., polyphenols, SARS-CoV-2, COVID-19, spike glycoprotein, neuropilin-1, in vitro, in silico

## Abstract

Since the outbreak of the COVID-19 pandemic, it has been obvious that virus infection poses a serious threat to human health on a global scale. Certain plants, particularly those rich in polyphenols, have been found to be effective antiviral agents. The effectiveness of *Alchemilla viridiflora* Rothm. (Rosaceae) methanol extract to prevent contact between virus spike (*S*)-glycoprotein and angiotensin-converting enzyme 2 (ACE2) and neuropilin-1 (NRP1) receptors was investigated. In vitro results revealed that the tested samples inhibited 50% of virus-receptor binding interactions in doses of 0.18 and 0.22 mg/mL for NRP1 and ACE2, respectively. Molecular docking studies revealed that the compounds from *A. viridiflora* ellagitannins class had a higher affinity for binding with S-glycoprotein whilst flavonoid compounds more significantly interacted with the NRP1 receptor. Quercetin 3-(6″-ferulylglucoside) and pentagalloylglucose were two compounds with the highest exhibited interfering potential for selected target receptors, with binding energies of −8.035 (S-glycoprotein) and −7.685 kcal/mol (NRP1), respectively. Furthermore, computational studies on other SARS-CoV-2 strains resulting from mutations in the original wild strain (V483A, N501Y-K417N-E484K, N501Y, N439K, L452R-T478K, K417N, G476S, F456L, E484K) revealed that virus internalization activity was maintained, but with different single compound contributions.

## 1. Introduction

Virus infections are now more clearly than ever a severe hazard to human health on a worldwide scale. SARS-CoV-2 triggered one of the deadliest pandemics in human history, with over 500 million confirmed cases of infection by worldometer (https://www.worldometers.info/coronavirus/) (accessed on 29 June 2022). The SARS-CoV-2 virus causes COVID-19 disease, which has a wide range of symptoms ranging from mild and asymptomatic cases to respiratory infections with fatal consequences. In addition to the deaths of over 6 million people worldwide, this pandemic imposed a new strain on all countries, causing local healthcare systems to collapse. Recent research studies have provided a detailed explanation of the SARS-CoV-2 virus’s entrance into the host cell [1]. This is a complex process since it requires multiple enzymatic structures from the host cell to be activated in stages. Coronavirus’ S glycoprotein is a structural component required for interaction with the host receptor. Previous research has shown that entry glycoproteins are typically split into two subunits before being internalized by the host cell. SARS-CoV-2 S glycoprotein is made up of two subunits: S1 is in charge of making contact with ACE2 and S2 attaches virus glycoprotein to the host cell’s membrane [2]. Then, this initiates a multi-step process that involves, furin convertase and transmembrane protease serine 2 [3]. However, it became obvious that an alternative method of internalization exists once it was shown that viruses may infiltrate host cells without using the ACE2 receptor. This method for virus internalization has recently been discovered to include neuropilin-1 receptors [4]. 

The anti-SARS-CoV-2 activity was studied in a number of medicinal plants with a documented history of antiviral use in traditional medicine [5]. One of their shared properties is the abundant presence of compounds with polyphenol chemical moiety. Plant polyphenols are a diverse group of molecules, and their substantial presence in plant tissue is associated with many medicinal plants’ health-beneficial properties (antioxidant, antidiabetic, antibacterial, etc.) [6,7]. It has been shown that phenolic compounds can block viral attachment to the human angiotensin-converting enzyme 2 (ACE2) receptor by interacting with the spike (S)-glycoprotein’s receptor-binding region [8]. One of the most promising modes of action for natural compounds has been identified as the interaction between S-glycoprotein and the ACE2 receptor [9]. In fact, numerous naturally occurring substances with known antiviral properties, such as hesperidin, punicalin, and punicalagin demonstrated potent anti-SARS-CoV-2 activity in a variety of in vitro and in silico studies. This was attributed to contact interference between the virus and the ACE2 receptor on a host cell [10,11,12,13].

**Figure 1:**
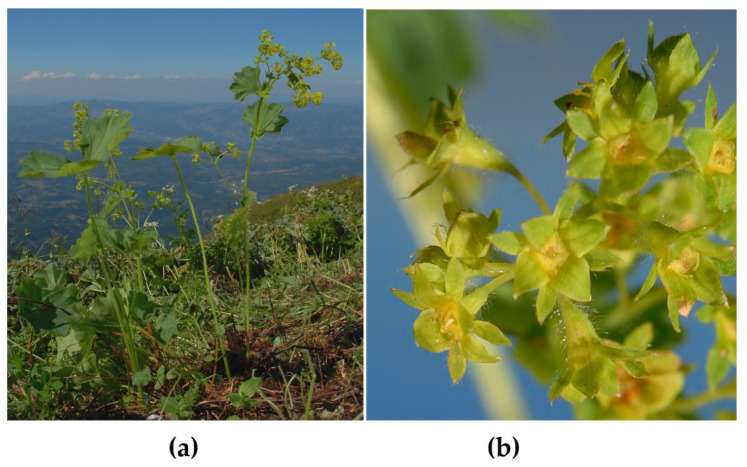
*Alchemilla viridiflora* Rothm: (**a**) plant at natural habitat; (**b**) magnified flowering parts.

The traditional medical usage of many *Alchemilla* species for treating viral infections has been supported by recent studies that demonstrated virucidal activity against influenza and orthopoxviruses [14,15]. Recent investigations have revealed that *Alchemilla* *viridiflora* Rothm. (Figure 1) polar extract possessed a strong ACE inhibitory effect, with particular components, such as miquelianin, being emphasized for their individual contributions to this activity [16]. 

This could be yet another link between *A. viridiflora* constituents and SARS-CoV-2 given that the incidence of COVID-19 disease requiring hospital admission is significantly reduced while taking ACE inhibitors [17]. Even though immunization is the most effective strategy to avoid SARS-CoV-2 infection, there are some circumstances where people are unable to get vaccinated due to medical reasons. Therefore, there is a need to develop alternative strategies to prevent and treat SARS-CoV-2 infection in these individuals. To avoid infection or at least reduce viral load, one strategy is to use natural compounds in appropriate pharmaceutical dosage forms to block early contact between the virus and ACE2 and NRP1 receptors.

To the best of the authors’ knowledge, *Alchemilla* isolates have not yet been investigated for their capacity to prevent SARS-CoV-2 infection despite being a rich source of bioactive polyphenols with demonstrated antiviral activity. The overall aim of this study is to clarify *A. viridiflora* methanol extract’s real potential for SARS-CoV-2 internalization through two main mechanisms by applying in silico and in vitro studies.

## 2. Results and Discussion

### 2.1. LC-MS Chemical Analysis (Phytochemical Analysis)

Given that polyphenols are thought to be the primary bioactive chemicals in *Alchemilla* species, the polyphenolic profile has been thoroughly examined. According to our previous study conducted on the same extract, 23% of the polyphenolic components of *A. viridiflora* are present in the dry methanol extract [16]. Similarly, ellagitannins and flavonoids are two of the most abundant polyphenol classes in the sample used in this study. A total of 17 compounds were identified using LC-MS analysis (mass spectra and chromatograms are shown in Figures S1 and S2 of the Supplementary Materials, respectively) of *A. viridiflora* methanol extract with subsequent MS data processed and analyzed using MestReNova software (Table 1). Pedunculagin, tellimagrandin I, tellimagrandin II and galloyl-bis-hexahydroxydiphenoyl (HHDP) hexose constituted the major ellagitannin fraction of *A. viridiflora* extract while flavonoid fraction comprised quercetin, quercetin derivates and kaempferol glycosides. The recent chemical characterization of *A. viridiflora* extract provided by Radovic et al. (2022) is consistent with the present phytochemical analysis [16].

Pedunculagin is a significant monomeric ellagitannin commonly found in *Alchemilla* species that has been related to various biological activities such as antitumor, antioxidant, gastroprotective, hepatoprotective, and anti-inflammatory properties [18]. Additionally, tellimagrandin I, a compound that was just recently identified as a constituent of *Alchemilla* species, and brevifolin carboxylic acid, an *Alchemilla* ellagitannin product of hydrolysis that is also typically found in various pomegranate parts, are both recognized for their bioactivity and significant antiviral activity [16,19,20]. Compounds from the flavonoid class are equally important in terms of biological function. Numerous activities, including those related to the prevention of SARS-CoV-2, have been linked to quercetin, its derivatives and isorhamnetin [21].

### 2.2. Molecular Docking Studies

To investigate the individual effects of the positively identified components of the tested *A. viridiflora* sample, we used molecular docking simulations. Starting with the most active compound, quercetin 3-(6″-ferulylglucoside), all compounds are listed in Table 2 in order of their binding affinity for the S-glycoprotein receptor (PDB ID: 7BZ5). This target’s binding pocket is depicted in Figure S3, and its constituent residues are given in Table S1 (Supplementary Materials).

**Figure 2:**
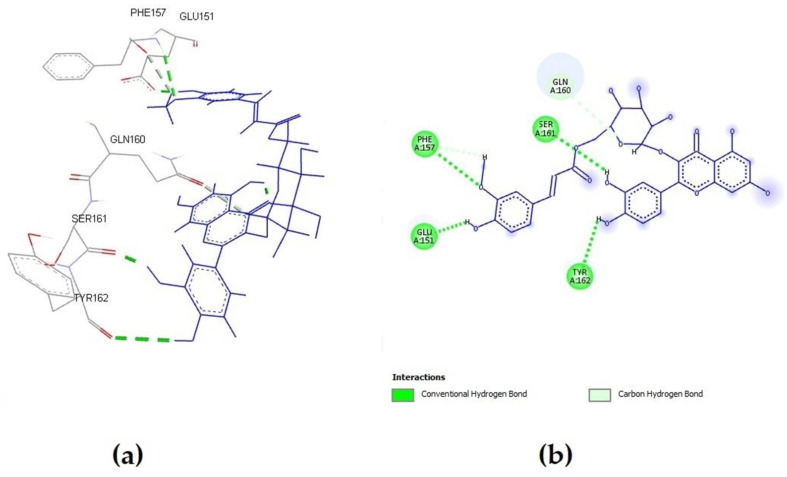
Quercetin 3-(6″-ferulylglucoside) interactions with S-glycoprotein (wild); (**a**) the most favorable binding pose of the compound; (**b**) 2D illustration of interaction types between the compound and target residues.

These findings demonstrated that the observed inhibitory activity was a result of contributions from both polyphenolic groups. Quercetin 3-(6″-ferulylglucoside) demonstrated the highest binding affinity (−8.035 kcal/mol). The most favorable binding orientation of this compound is presented in Figure 2. Preliminary 12.50 ns molecular dynamic simulation results for the quercetin 3-(6″-ferulylglucoside)-S-glycoprotein complex presented in Figures S4–S7 confirm the stability of the observed system. Radius gyration trajectory (Figure S5) deviations between 18.40 Å and 18.85 Å indicate a stable secondary protein structure with a high complexing potential for the studied ligand. Observing the root mean square deviation (RMSD) trajectory (Figure S6) reveals that after 2 ns, the complex reaches a stable state. The complex exhibits simulation-based deviations after that point that do not compromise the system’s stability because oscillations between the mean and maximum value do not exceed 2.5 Å.

**Figure 3:**
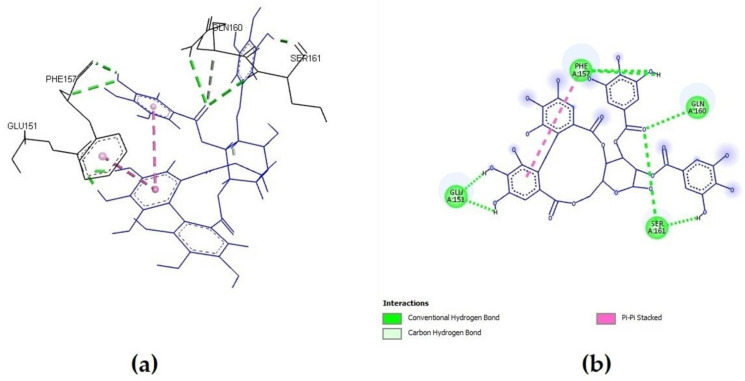
Tellimagrandin I interactions with S-glycoprotein (wild): (**a**) the most favorable binding pose of the compound; (**b**) 2D illustration of interaction types between the compound and target residues.

Two compounds from the ellagitannin class, tellimagrandin I and II, showed only a somewhat lower affinity for interacting with S-glycoprotein, with binding affinity energies of −8.022 and −7.955 kcal/mol, respectively. Additionally, every compound that was tested produced complexes with the target protein that were stabilized by regular hydrogen bonds on distances lower than 2 Å. One of the key interacting residues, Gln160, was previously identified as one of 23 virus residues that participate in stable hydrogen bonds, which let the virus bind to the ACE2 receptor. Most of the tested *A. viridiflora* polyphenols showed interaction with this residue, and the second-most potent compound, tellimagrandin I, interacted with it via an H bond at a distance of 2.82 Å (Figure 3) [22]. 

When complexing with the target S-glycoprotein, the two positive controls utilized in this investigation showed a very slight energy difference, with umifenovir forming a more stable complex (−7.384 kcal/mol) than quercetin (−7.189 kcal/mol).

According to a recent study, umifenovir inhibits the internalization of SARS-CoV-2 and its variants by directly binding to the S-glycoprotein {Shuster, 2021 #41}. However, 12 polyphenolic *Alchemilla* constituents exhibited more affinity for S-glycoprotein as a target than umifenovir, indicating more effective infection prevention (Table 2).

**Figure 4:**
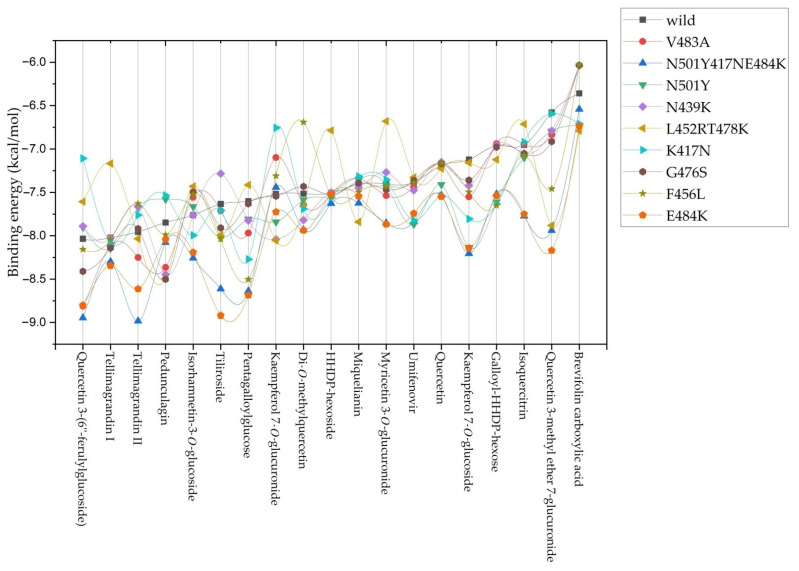
Binding energy (kcal/mol) curves for *A. viridiflora* constituents and positive controls against all tested S-glycoprotein structural variants.

In addition to docking against wild strain-specific S-glycoprotein more docking simulations were performed on the V483A, N501Y417NE484K, N501Y, N439K, L452RT478K, K417N, G476S, F456L, and E484K strains to evaluate the stability of the observed inhibitory action on other viral strains resulting from mutations. The binding energy fluctuations curve for S-glycoprotein revealed that different drugs had varying binding affinities. In particular, the mutated strains identified in South Africa lineage B.1.351 (also known as 501Y.V2 variant) and P.1 lineage (a descendant of B.1.1.28) identified in December 2020 (in Manaus, Amazonas State, North Brazil) showed increased affinity for quercetin and tellimagrandin II, compounds with binding affinity on first and third place for wild type virus S-glycoprotein (Figure 4) [23,24]. The increased binding affinity seen in Figure 4 for the positive control umifenovir is also consistent with the findings of Shuster et al. (2021) about its maintained activity against new virus strains [25].

Although variations in binding affinity were observed for all identified constituents of *A. viridiflora* extract overall conclusion is that the range of the complex energies between −6.0 and −9.0 kcal/mol for all compounds proves they maintained significant inhibitory potential regardless of mutation changes in S-glycoprotein. According to these results, the tested extract should retain its efficacy against other virus strains, which is necessary given the significant mutational potential identified for the SARS-CoV-2 virus.

**Figure 5:**
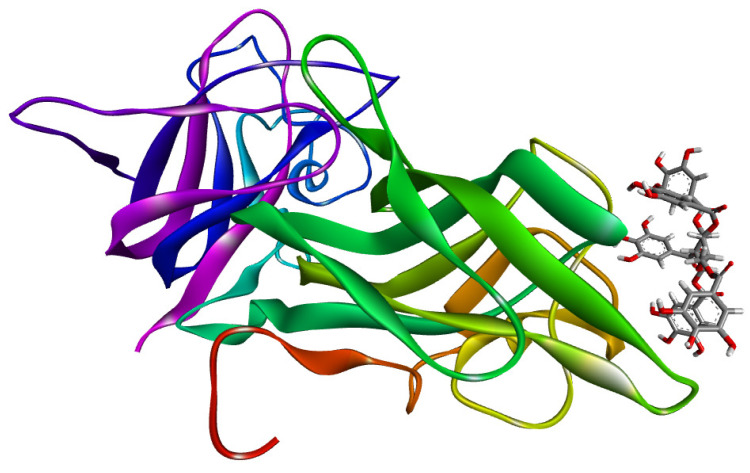
Pentagalloylglucose binding pose and site in complex with NRP1.

Molecular docking simulation results of *A. viridiflora* constituents and positive control against the NRP1 target are presented in Table 3. Seven compounds displayed a greater affinity for NRP1 than the positive control brevifolin carboxylic acid, showing that other polyphenols also significantly contribute to the inhibitory activity. Figure 5 presents the binding position and active site of pentagalloylglucose (compound with the highest binding activity) interaction with NRP1. 

In Jin et al. study from 2022, pentagalloylglucose, the molecule listed first for its affinity to NRP1, was already recognized as a plant dietary polyphenol with substantial in vitro inhibitory effect against SARS-CoV-2 infection in Vero cells. This study confirmed that part of this inhibitory potential could be attributed to the inhibition of SARS-CoV-2 main- and RNA-dependent RNA-polymerase. Additionally, researchers found efficacy against the SARS-CoV and MERS-CoV viruses, indicating pentagalloylglucose has broad-spectrum anticoronaviral potential [26].

Other compounds with energy values below −6.5 kcal/mol, apart from pedunculagin, were from the flavonoid class, indicating that the flavonoid subclass of *A. viridiflora* polyphenols may contribute more significantly to inhibition activity when the virus primarily uses the NRP1 receptor for its internalization. Flavonoid potential for interaction with NRP1 has been already discussed in Yasmin et al. (2017) study where they proposed quercetin and diosmin as small ligands with promising potential for targeting NRP1 receptors with implications for therapeutic benefits in neurology and oncology [27]. Multiple interaction types contribute to the stabilization of ligand-target complexes, and it is noteworthy that all tested ligands were stabilized by at least one conventional hydrogen bond at a distance closer to 3 Å. The pharmacophore model highlighted ligand interactions with NRP1 residues: Tyr353, Thr349, Tyr297, Asn300, and Ser298 as critical for inhibitory activity in the Perez-Miller et al. (2020) investigation that found and confirmed inhibitors of the interaction between NRP1 and SARS-CoV-2 S-glycoprotein [28]. All these significant NRP1 residue interactions are also identified in the most favorable binding poses of *A. viridiflora* polyphenol constituents. In addition to H bonds with Tyr353, pedunculagin interacts with the same type of interaction with Asp320, a crucial residue for interaction with vascular endothelial growth factor C-terminal arginine [29]. In addition to conventional H-bond interactions, ligands, particularly those with a flavonoid structure, were stabilized by hydrophobic interactions. These interactions included ring B from the flavonoid structure and the amino acid residues Tyr297, Thr316, Tyr353 and Trp411 from the binding pocket presented in Figure S7.

### 2.3. In Vitro SARS-CoV-2 Internalization Inhibition Assays

**Figure 6:**
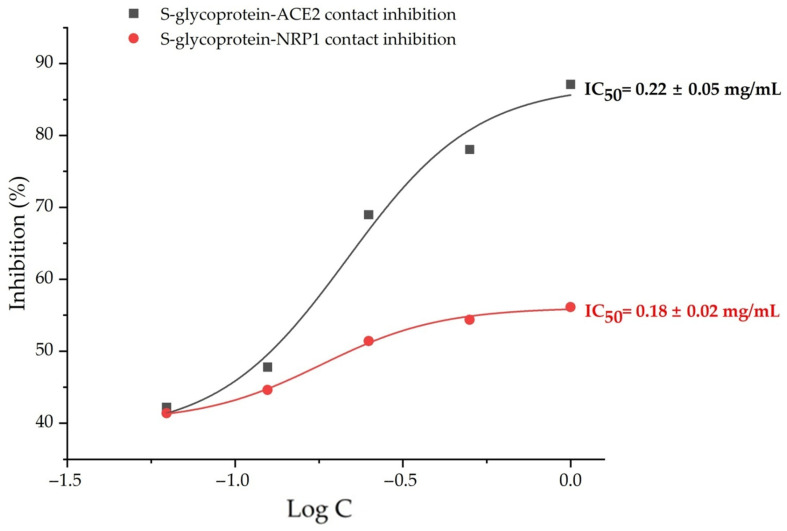
Concentration-inhibition curves of *A. viridiflora* methanol extract on S-glycoprotein-ACE2 (black line) and S-glycoprotein-NRP1(red line) contacts.

The binding inhibitory effects of an extract of *A. viridiflora* on S glycoprotein-ACE2 and S glycoprotein-NRP were explored as a mechanism of their anti-SARS-CoV-2 potential in this work. Umifenovir and quercetin served as positive controls for the S glycoprotein-ACE2 inhibition assay, and brevifolin carboxylic acid served as a positive control for the S glycoprotein-NRP inhibition assay, both at the same concentrations as the samples. The extract was tested at concentrations ranging from 0.0625 to 1.00 mg/mL. The results indicated that the tested *A. viridiflora* extract was able to inhibit S-glycoprotein interactions with both receptor targets in a dose-dependent manner. The inhibition values for S-glycoprotein binding to NRP1 and ACE2 were 56.3% and 87.1%, respectively, at the highest tested concentration of *A. viridiflora* methanol extract (1.00 mg/mL). Positive controls umifenovir and quercetin inhibited S-glycoprotein and ACE2 contact at the highest tested concentrations (1 mg/mL), with inhibition values of 5.22% and 2.10%, respectively, whereas brevifolin carboxylic acid at the same concentration inhibited contact between S-glycoprotein and NRP1 by 63.07%. These in vitro results are consistent with the docking study simulation, which indicated that umifenovir had a higher inhibtion potential than quercetin, another positive control and that *A. viridiflora* methanol extract could have an even more potent antiviral effect considering 12 constituents with a higher affinity for the same target than umifenovir. Brevifolin carboxylic acid suppressed S-glycoprotein and NRP1 interactions more effectively than *A. viridiflora* extract, whose constituent it is. Possible cause could be its lower concentration in extract relative to less potent polyphenols. Using the OriginPro v. 9.8.0.200 program (OriginLab Corp.), the doses that resulted in a 50% inhibition of binding interactions between S-glycoprotein and receptors for internalization were determined to be 0.18 and 0.22 mg/mL for NRP1 and ACE2, respectively (Figure 6).

Results obtained in vitro are consistent with recently published research that investigated the potential of pomegranate ellagitannin-rich extracts to inhibit the interaction between S-glycoprotein and ACE2 [11,13]. Ellagitannin polyphenols from pomegranate peel extract synergistically inhibited contact between virus S-glycoprotein and ACE2 receptor. Tellimagrandin I and brevifolin carboxylic acid, two of the ellagitannins found in *A. viridiflora*, are frequently present in different pomegranate extracts. The significant potential of *A. viridiflora* extract for inhibiting SARS-CoV-2 internalization via ACE2 receptors may be explained by these complementary ellagitannins and other compounds from the same class [16,30]. Additionally, it was discovered that urolithin A, a common ellagitannin metabolite in humans, is a potent inhibitor of SARS-CoV-2 binding to the ACE2 receptor [13]. Liu et al. (2020) in their in vitro study demonstrated that quercetin, *A. viridiflora* major flavonoid representative has potency for the recombinant human ACE2 receptor inhibition, at physiologically relevant dosages [31]. This is a further strong indication that two classes of polyphenols have synergistic inhibitory effects on the internalization of SARS-CoV-2 through the ACE2 host receptor. However, the study’s findings donot support a firm conclusion in this way and instead can serve as the foundation for some additional investigation. In order to determine whether NRP1 was a host factor for SARS-CoV-2, Daly et al. (2020) employed the small ligand EG00229. This ligand was a confirmed NRP1 antagonist and it was shown to be bound to NRP1 with a Kd of 5.1 and 11.0 μM at pH 7.5 and 5.5, respectively [32]. It was also previously determined that EG00229 in 3 μM concentration selectively inhibits 50% of vascular growth endothelial factor A binding to a purified NRP1 b1 domain [33]. However, despite doing a thorough literature search, the authors were unable to find any additional studies that examined a natural small ligand as an NRP1 antagonist.

## 3. Materials and Methods

### 3.1. Plant Material and Extract Preparation

Plant material of *Alchemilla viridiflora* Rothm., Rosaceaeand the preparation of the methanol extract was previously described by Radovic et al. (2022), and the same extract was used to conduct the current study [16]. Briefly, *A. viridiflora***,** aerial flowering parts were collected on carbonate soil in subalpine pastures at 1750 m s.m. on Mount Suva Planina in July 2019. Dr. Marjan Niketic identified plant material from voucher specimens (20130708/1-2) that were placed in the Natural History Museum (Belgrade, Serbia). The dry extract was obtained by methanol extraction for two days after which the solvent was evaporated under low pressure. The yield of dry methanol extract from powdered plant material was 28.3%. 

### 3.2. Chemicals

Analytical grade methanol was obtained from Macron Fine Chemicals (Avantor, Radnor, PA, USA); analytical grade dimethyl sulfoxide was obtained from FisherScientific (Fair Lawn, NJ, USA).Acetonitrile and formic acid for HPLC, gradient grade were purchased from Sigma-Aldrich (St. Louis, MO, USA). Arbidol hydrochloride (umifenovir) for HPLC (≥98%) and quercetin for HPLC (≥95%) reference standard were purchased from Sigma-Aldrich (St. Louis, MO, USA). Brevifolin carboxylic acid for HPLC (≥95%) reference standard was purchased from Cayman Chemical (Ann Arbor, MI, USA). 

### 3.3. LC-MS Chemical Analysis 

The LC-MSanalysis of *A. viridiflora* methanol extract was performed and described in our earlier research, with an addition in this study relating to the software used for the identification of individual compounds [16]. Agilent Technologies HPLC1260 Infinity system connected to a single quadrupole mass detector (Singlequad MS detector 6130) was employed.Compound separation was carried out at 25 °C using a Zorbax SB Aq-C18 column (3.0 × 150 mm; 3.5 µm). Solvent A (0.1% HCOOH in water) and Solvent B (acetonitrile) were used for elution.With a flow rate of 0.3 mL/min, the gradient program listed below was used: 0–30 min from 10% to 25% B; 30–35 min from 25% to 70% B; 35–40 minreturn to 10% B. Detection wavelengths were at 280 and 350 nm and in negative mode in a range of 50–2000 *m*/*z*. Electrospray ionization was carried out at a pressure of 40 psi, a temperature of 350 °C, and a nitrogen flow rate of 10 L/min. Signals from deprotonated molecules and fragmented ions were acquired in full-scan at fragmentation voltages of 100 V and 250 V. MestReNova v.12.0.0-20080 (Mestrelab Research, S.L., Santiago de Compostela, Spain) software’smolecule match tool was used for identification of compounds instead of tentative identification based on comparison with literature data, as it was performed in our previous study on this extract [16]. 

### 3.4. Molecular Docking Simulations

#### 3.4.1. Dataset

For the first target, the crystal structure of S-glycoprotein RBD in a complex with neutralizing body was retrieved from the Protein Data Bank (PDB; http://www.pdb.org, PDB ID:7BZ5) and prepared for docking by DockThor-VS web server as a new COVID-19 resources service (accesed and submitted on 11 May 2022). Besides the wild type S glycoprotein, docking studies were conducted on 9 mutation variants (V483A, N501Y-K417N-E484K, N501Y, N439K, L452R-T478K, K417N, G476S, F456L, E484K) of the same structure (PDB ID:7BZ5) [34]. For the second target, the crystal structure of the b1b2 domains from human neuropilin-1 (PDB ID:2QQI) was retrieved from PDB and prepared for the docking analysis using Yasara Structure (v. 20.4.24.W.64, YASARA Biosciences GmbH, Vienna, Austria) (http://www.yasara.org/; accessed on 11 May 2022). This procedure included deletion of solvents from the PDB files, adding hydrogens and charges to the structure, and the process of energy minimization. The 3D molecular structures of identified polyphenols and positive controls were downloaded from PubChem (https://pubchem.ncbi.nlm.nih.gov/; accessed on 11 May 2022) whereas compounds without 3D structures were downloaded as 2D structures and after that converted into 3D structures via online service (http://pccdb.org/tools/convert_3D_mol; accessed on 11 May 2022). All ligands’ final geometries were energy minimized using the Yasara Structure energy minimization experiment option using AMBER03 force field at physiological pH (7.4), which ran local steepest descent minimization without electrostatics to eliminate bumps, followed by simulated annealing minimization energy with a certain energy improvement.

#### 3.4.2. Docking Parameters

Molecular docking simulations for the first target (PDB ID:7BZ5) were conducted inside a 20 Å size cubic grid box which was centered around Cα of Gln493 residue located at the binding zone of S glycoprotein and ACE-2 residues. For the second target (PDB ID:2QQI) grid box was generated around Asp320, Ser346, Thr316, Thr349 and Tyr353 residues within a distance of 5 Å. The docking procedure was conducted through Yasara Structure software based on the AutoDockVina algorithm and AMBER03 force field [35]. Output files of the most stable complexes were further analyzed with the visualization software (Discovery Studio Visualizer v.20.1.0.19295, Dassault Systèmes, Vélizy-Villacoublay, France).

### 3.5. Molecular Dynamics (MD) Simulation

Preliminary MD simulation for the energetically most favored quercetin 3-(6″-ferulylglucoside)-S-glycoprotein complex (determined by docking simulations) was conducted using YASARA Structure v. 20.12.24.W.64. Hydrogen (H)-bond optimization and pKa prediction for the chosen pH (7.4) were part of the experimental setup [36]. The addition of NaCl ions (0.9%, cell neutralization, and energy minimization provided the correct structure’s geometry. The MD simulation was run for 12.50 ns with the AMBER14 force field. The setup used 298 K and one atmosphere for temperature and pressure values, respectively. The composition of the simulated system is given in Table S2.

### 3.6. In Vitro SARS-CoV-2 Internalization Inhibition Assays

To investigate the in vitro effects of *A. viridiflora* polyphenols on SARS-CoV-2 binding activity to ACE2 and NRP1 the MBS669459 screening kit (https://www.mybiosource.com/covid-19-assay-kits/covid-19-coronavirus/669459 accessed on 29 June 2022) and RayBio COVID-19 Spike-NRP1 Binding assay kit (https://doc.raybiotech.com/pdf/Manual/CoV-NRP1S1_2021.10.06.pdf accessed on 29 June 2022) were employed. Both assays were based on a colorimetric ELISA kit that measures the binding of RBD of the S-glycoprotein from SARS-CoV-2 (wild strain) to its human receptors ACE2 and NRP1, respectively. All tested samples were dissolved in phosphate buffer solution or DMSO the final concentration of which did not exceed 0.1%. Reagents preparation and assay procedure steps were conducted strictly following the provided protocols for the default configuration.

## 4. Conclusions

The results of in vitro research, as well as in silico, showed that methanol extract of *A. viridiflora* and its components were capable of considerably inhibiting the internalization of SARS-CoV-2 through two of its currently most significant receptors. Ellagitannins more clearly blocked S-glycoprotein’s interactions with ACE2, whilst flavonoids showed more affinity for interactions with the NRP1 receptor. Additionally, the structural changes to the S-glycoprotein brought on by mutations had a minor impact on the *A. viridiflora* constituens’ activity. Lastly, the polyphenols found in the methanol extract of *A. viridiflora* offer intriguing starting points for future in vitro and in vivo anti-SARS-CoV-2 research, particularly considering their potential synergistic activity.

## Figures and Tables

**Table 1:** Polyphenols identified in *A. viridiflora* methanol extract sample by LC-MS method.

Compound	Formula:	Molecular Weight:	Match Score:	RT:	Adduct/Loss:
Pedunculagin	C_34_H_24_O_22_	784.076	0.998	9.35	−/H+
Galloyl-HHDP hexose	C_27_H_22_O_17_	618.086	0.999	12.41	Na+/−
Isoquercitrin	C_21_H_20_O_12_	464.095	0.999	12.44	H+/−
Quercetin 3-(6″-ferulylglucoside)	C_31_H_28_O_15_	640.143	0.993	12.46	−/H+
Tellimagrandin I	C_34_H_26_O_22_	786.092	0.993	16.4	−/H+
Brevifolin carboxylic acid	C_13_H_8_O_8_	292.022	0.997	21.5	−/H_2_OH+
Myricetin 3-*O*-glucuronide	C_21_H_18_O_14_	494.07	0.973	22.9	CH_3_OHH+/−
Tellimagrandin II	C_41_H_30_O_26_	938.103	0.992	23.97	−/H+
Pentagalloylglucose	C_41_H_32_O_26_	940.118	0.879	29.36	−/H+
Kaempferol 7-*O*-glucuronide	C_21_H_18_O_12_	462.08	0.996	30.97	Na+/−
HHDP-hexoside	C_20_H_18_O_14_	482.07	0.961	31.1	CH_3_OHH+/−
Quercetin 3-methyl ether 7-glucuronide	C_22_H_20_O_13_	492.09	0.985	31.13	−/H+
Kaempferol 7-*O*-glucoside	C_21_H_20_O_11_	448.101	0.981	33.06	Na+/−
Di-*O*-methylquercetin	C_17_H_14_O_7_	330.074	0.999	33.82	−/H+
Tiliroside	C_30_H_26_O_13_	594.137	0.996	37.7	−/H+
Isorhamnetin-3-*O*-glucoside	C_22_H_22_O_12_	478.111	0.963	39.37	NH_4_+/−
Miquelianin	C_21_H_18_O_13_	478.075	0.96	39.37	NH_4_+/−

**Table 2:** Molecular docking simulation results of *A. viridiflora* constituents and positive controls against wild type S-glycoprotein target (PDB ID: 7BZ5).

Compound	Bind Energy [kcal/mol]	Interacting Residues *
Quercetin 3-(6″-ferulylglucoside)	−8.035	Gln160, **Glu151** (1.63 Å), **Phe157** (2.63 Å), **Ser161** (1.84 Å), **Tyr 162** (2.83 Å)
Tellimagrandin I	−8.022	**Gln160** (2.82 Å), **Glu151** (1.57 Å, 1.71 Å), **Phe157** (2.82 Å, 2.84 Å)
Tellimagrandin II	−7.955	Gln160, **Glu151** (1.59 Å, 1.64 Å), **Gly163** (3.10 Å), Tyr116, **Tyr116** (1.73 Å, 1.94 Å), **Tyr162** (2.24 Å)
Pedunculagin	−7.848	**Gln160** (2.23 Å), **Glu151** (1.64 Å, 2.15 Å), **Gly163** (2.70 Å), Leu119, Phe157, **Tyr116** (1.69 Å), **Tyr162** (2.46 Å)
Isorhamnetin-3-*O*-glucoside	−7.761	**Glu151** (1.63 Å, 1.76 Å), Leu119, **Leu159** (1.60 Å), Phe157, **Ser161** (2.92 Å), **Tyr162** (2.79 Å)
Tiliroside	−7.633	Arg70, **Gln73** (1.50 Å), Gln160, **Glu151** (1.61 Å), **Lys84** (2.73 Å), **Phe157** (2.42 Å), Tyr120
Pentagalloylglucose	−7.601	**Gln160** (2.52 Å), Gln160, **Glu151** (1.54 Å, 1.62 Å), **Ser161** (2.64 Å), Tyr156, **Tyr162** (2.02 Å)
Kaempferol 7-*O*-glucuronide	−7.519	**Glu151** (1.92 Å), **Phe157** (2.43 Å), **Tyr120** (1.20 Å)
Di-*O*-methylquercetin	−7.515	**Gln160** (2.38 Å), **Glu151** (1.59 Å, 1.60 Å), Phe123, **Phe157** (2.33 Å), Tyr156
HHDP-hexoside	−7.506	**Glu151** (1.71 Å, 2.00 Å), Phe157, **Ser161** (2.39 Å), **Tyr116** (2.34 Å)
Miquelianin	−7.406	**Gln160** (3.09 Å), **Glu151** (1.72 Å, 1.92 Å), Leu119, Phe157, **Ser161** (1.49 Å)
Myricetin 3-*O*-glucuronide	−7.404	**Glu151** (1.67 Å, 1.94 Å), Leu119, **Leu159** (1.73 Å), Phe157, **Ser161** (1.46 Å)
Umifenovir **	−7.384	**Glu151** (1.65 Å), **Ser161** (1.96 Å), Tyr116
Quercetin **	−7.189	**Gln160** (2.07 Å), **Glu151** (1.63 Å, 1.78 Å), Phe123, **Phe157** (2.02 Å), Tyr156
Kaempferol 7-*O*-glucoside	−7.121	**Gln160** (2.48 Å, 3.02 Å), **Glu151** (1.66 Å, 1.70 Å), **Phe157** (1.97 Å), **Tyr162** (1.90 Å)
Galloyl-HHDP hexose	−6.964	**Gln160** (2.56 Å, 2.77 Å), **Glu151** (1.56 Å, 1.82 Å), **Leu159** (1.70 Å, 1.96 Å)
Isoquercitrin	−6.953	**Gln160** (3.03 Å), **Glu151** (1.63 Å, 1.64 Å), **Leu159** (1.89 Å), **Ser161** (1.80 Å, 2.32 Å)
Quercetin 3-methyl ether 7-glucuronide	−6.579	**Glu151** (1.58 Å, 1.62 Å), **Lys84** (3.06 Å), Lys84, **Tyr120** (1.62 Å)
Brevifolin carboxylic acid	−6.359	**Arg70** (1.53 Å), **Tyr162** (1.84 Å), **Tyr172** (1.63 Å)

* In the interacting residues column residues involved in hydrogen bonding are denoted in bold font with the interaction distances enclosed in brackets. ** Positive control compounds are bordered with frame.

**Table 3:** Molecular docking simulation results of *A. viridiflora* constituents and positive control against NRP1 target (PDB ID: 2QQI).

Compound	The Most Favorable Binding Pose **	Bind Energy [kcal/mol]	Interacting Residues *
Pentagalloylglucose	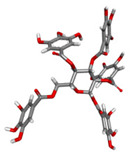	−7.685	**Asp320** (2.29 Å, 2.50 Å), **Lys351** (2.29, 2.32 Å), Lys351, **Lys352** (2.04 Å), **Pro317** (2.50 Å), **Thr413** (2.61 Å, 2.68 Å), Tyr297, **Tyr353** (2.41 Å, 2.71 Å)
Quercetin methyl ether glucuronide	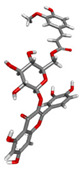	−7.667	**Asp320** (2.72 Å), **Glu348** (2.09 Å), **Lys351** (2.07 Å), Thr413, **Thr413** (3.01 Å), **Trp301** (2.00 Å), Trp411,Tyr297, **Tyr353** (2.51 Å)
Tiliroside	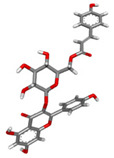	−7.594	Asp320, **Glu348** (2.15 Å), **Lys351** (2.05 Å), Thr413, Tyr297, **Tyr353** (2.82 Å)
Kaempferol 7-*O*-glucuronide	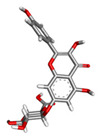	−7.452	**Asn300** (1.82 Å), Asp320, **Lys351** (2.05 Å), **Trp301** (2.36 Å), Tyr297
Kaempferol 7-*O*-glucoside	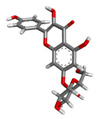	−7.264	**Asn300** (1.85 Å), Asp320, **Lys351** (2.02 Å), **Trp301** (2.30 Å), Tyr297
Quercetin	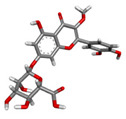	−7.205	**Asn300** (2.60 Å), Asp320, **Lys351** (1.94 Å, 2.85 Å), **Thr349** (2.72 Å), **Trp301** (2.28 Å), **Tyr297** (2.51 Å), Tyr353
Miquelianin	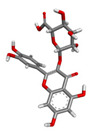	−6.986	**Asp320** (2.44 Å), **Glu348** (2.65 Å), **Lys351** (1.98 Å), **Thr349** (2.60 Å), Thr413
Brevifolin carboxylic acid ***	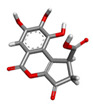	−6.976	**Lys351** (2.00 Å), Thr316, **Trp301** (2.32 Å), Trp301, Tyr297
Quercetin 3-(6″-ferulylglucoside)	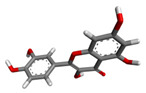	−6.875	**Arg418** (2.65 Å), Arg418, **Asn309** (2.90 Å), **Asn313** (2.37 Å),Glu312, Ile345, **Lys350** (2,84 Å), **Ser346** (2.83 Å), Thr388
Pedunculagin	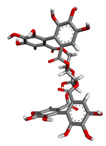	−6.855	**Asn300** (2.78 Å), Asp320, Ser298, **Tyr297** (2.33 Å, 2.49 Å)
Myricetin 3-*O*-glucuronide	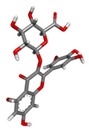	−6.802	Asp320, **Gly318** (2.17 Å), **Lys351** (1.99 Å), **Thr349** (2.72 Å),Thr413, Tyr297
Isoquercitrin	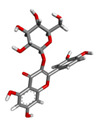	−6.799	Asp320, **Gly318** (2.52 Å), **Lys351** (2.03 Å, 2.52 Å), **Ser346** (3.02 Å), Thr413, Tyr297
Tellimagrandin I	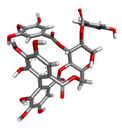	−6.76	Asp320, **Glu319** (2.77 Å), Gly318, Ser321, **Thr413** (1.93 Å), Tyr297
Di-*O*-methylquercetin	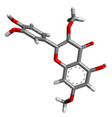	−6.713	**Asn300** (2.05 Å), Glu348, Tyr297, Tyr301, Tyr353
Isorhamnetin-3-*O*-glucoside	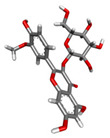	−6.467	Asp320, Asp320 (2.71 Å), Gly318, **Lys351** (2.25 Å, 2.40 Å), **Ser346** (2.65 Å), Thr413, **Thr413** (2.56 Å), Tyr297
Galloyl-HHDP-hexose	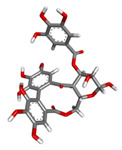	−6.365	Asp320, **Asp320** (2.07 Å, 2.92 Å), Thr413, Trp301, **Trp411** (2.51 Å), Tyr297, **Tyr353** (2.67 Å)
Tellimagrandin II	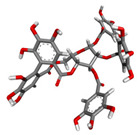	−6.051	Arg323, **Asp320** (2.76 Å), Tyr297
HHDP-hexoside	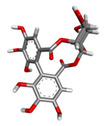	−5.897	**Lys351** (2.02 Å), **Thr413** (2.46 Å), Tyr353

* In the interacting residues column residues involved in hydrogen bonding are denoted in bold font with the interaction distances enclosed in brackets. ** 3D structure atoms color legend; red-oxygen, grey-carbon, light grey-hydrogen. *** positive control compound is bordered with frame.

## Data Availability

All data are available upon reasonable request.

## Supplementary Materials

The following supporting information can be downloaded at: https://www.mdpi.com/article/10.3390/molecules27165174/s1. Figure S1: *A. viridiflora* methanol extract mass spectrum; Figure S2: *A. viridiflora* methanol extract base peak chromatogram; Figure S3: Graphical presentation of binding pockets with constituent amino acid residues (ball and stick display style) for protein targets used in docking simulations (a) S-glycoprotein (PDB:7BZ5) with binding pocket residues marked red; (b) Neuropilin-1 (PDB:2QQI) with binding pocket residues marked blue; Figure S4: A ray-traced picture of the simulated system. The simulation cell boundary is set to periodic; Figure S5: Total potential energy of the system [vertical axis] as a function of simulation time [horizontal axis]; Figure S6: Radius of gyration of the solute [vertical axis] as a function of simulation time [horizontal axis]; Figure S7: Ligand movement root mean square deviation (RMSD) after superposing on the receptor [vertical axis] as a function of simulation time [horizontal axis]; Table S1: Binding pocket residues list for protein targets used in docking simulations; Table S2: Composition of the simulated system.
