# Supplementary material for: In Silico and In Vitro Studies of Alchemilla viridiflora Rothm—Polyphenols’ Potential for Inhibition of SARS-CoV-2 Internalization
Source: Molecules. 2022 Aug 14;27(16):5174. doi: 10.3390/molecules27165174 (PMC9415016; doi:10.3390/molecules27165174)
Supplement: Supplementary file 1 [file molecules-27-05174-s001.zip › molecules-1816542-supplementary.pdf]

Supplementary materials

1. Chemical analysis

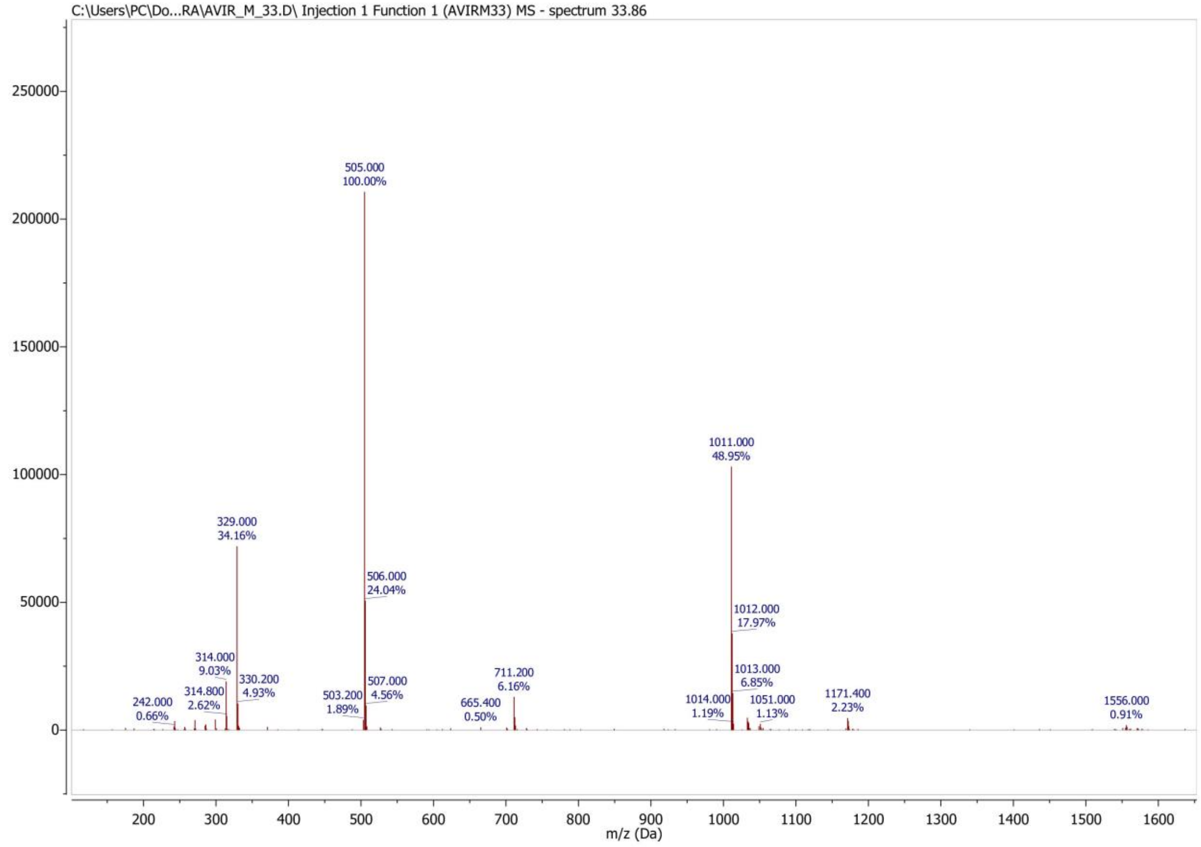

Figure S1. *A. viridiflora* methanol extract mass spectrum

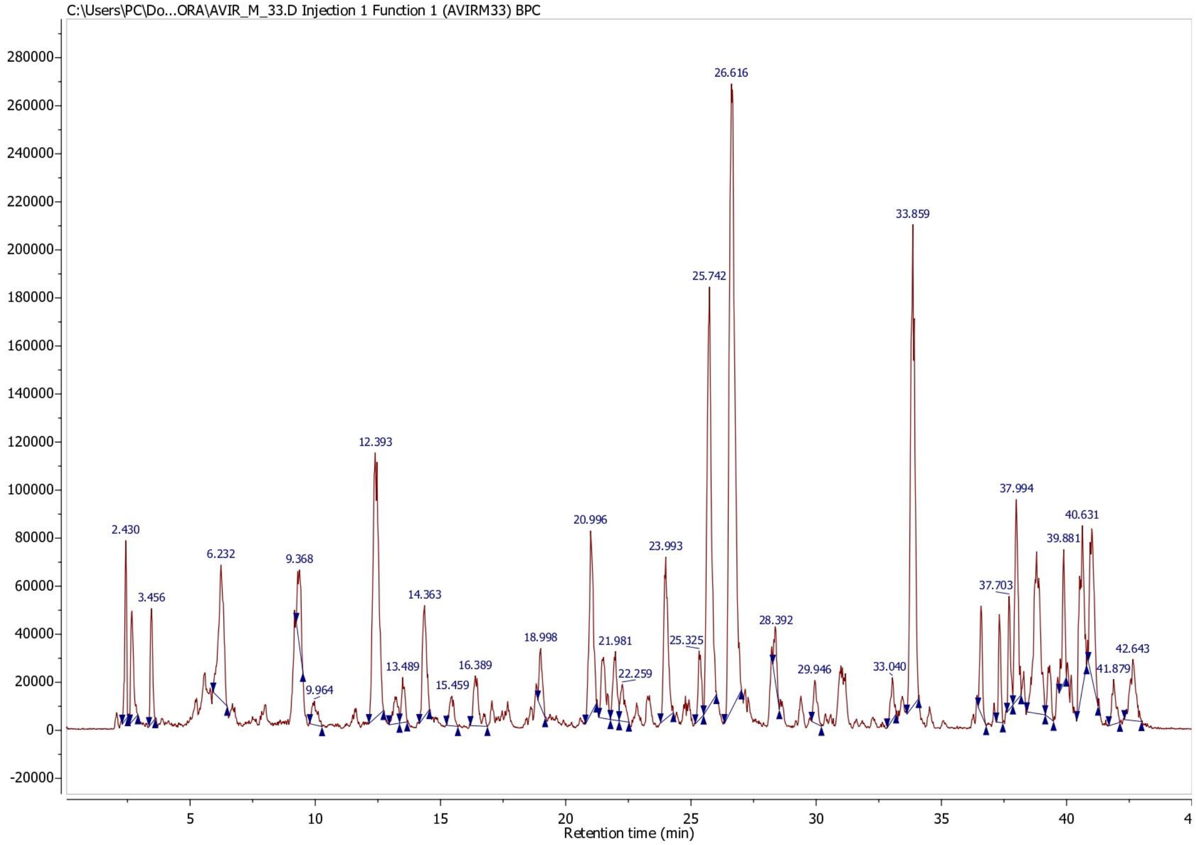

Figure S2. *A. viridiflora* methanol extract base peak chromatogram

2. Molecular docking simulations

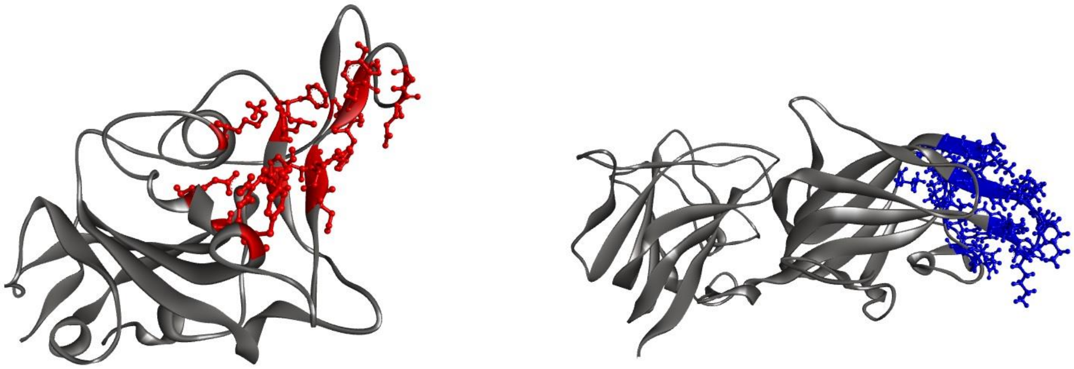

(a) (b)

Figure S3. Graphical presentation of binding pockets with constituent amino acid residues (ball and stick display style) for protein targets used in docking simulations (a) S-glycoprotein (PDB:7BZ5) with binding pocket residues marked red ; (b) Neuropilin-1 (PDB:2QQI) with binding pocket residues marked blue

Table S1. Binding pocket residues list for protein targets used in docking simulations

Spike (PDB:7BZ5)	Neuropilin-1 (PDB:2QQI)
Arg70	Tyr297
Glu73	Ser298
Lys84	Asn300
Tyr120	Trp301
Leu122	Asn313
Phe123	Thr316
Ile139	Pro317
Glu151	Gly318
Gly152	Glu319
Cys155	Asp320
Tyr156	Ser321
Phe157	Glu324
Leu159	Ile345
Gln160	Ser346
Ser161	Lys347
Tyr172	Glu348
	Thr349
	Lys350
	Lys351

	Lys352
	Tyr353
	Tyr354
	Trp411
	Glu412
	Thr413
	Gly414
	Ile415
	Ser416

3. Molecular dynamic simulations

The trajectory of Quercetin 3-(6''-ferulylglucoside) in complex with S-glycoprotein (PDB ID: 7BZ5) has been analyzed with YASARA version 20.12.24.W.64 over a period of 12.50 nanoseconds with 51 snapshots and the AMBER14 force field.

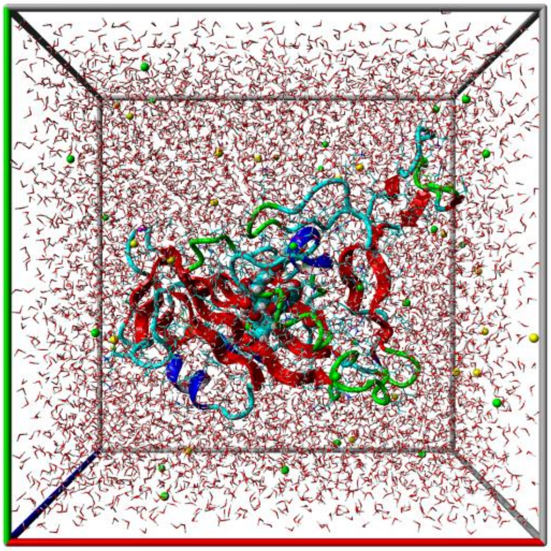

Figure S4. A ray-traced picture of the simulated system. The simulation cell boundary is set to periodic

Table S2. Composition of the simulated system

Type	Number
Protein molecules	1
Protein residues	194

Protein atoms	2998
Nucleic acid molecules	0
Nucleic acid residues	0
Nucleic acid atoms	0
Residue LIG with 74 atoms	1
Element Cl	26
Element Na	23
Water residues	8965
Total number of atoms	30016

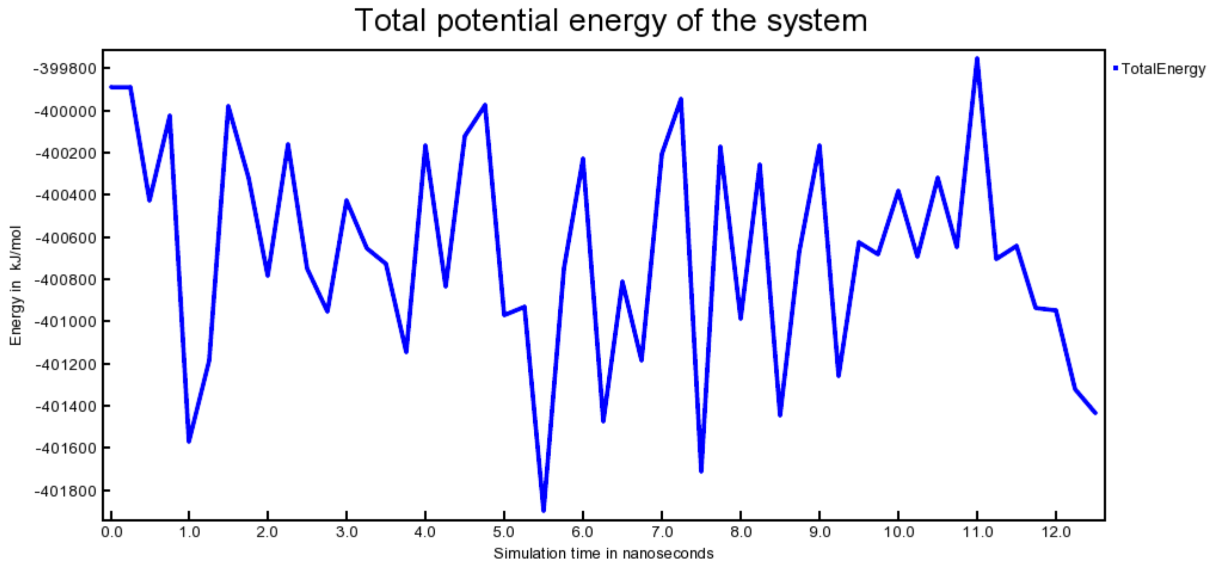

Figure S5. Total potential energy of the system [vertical axis] as a function of simulation time [horizontal axis]

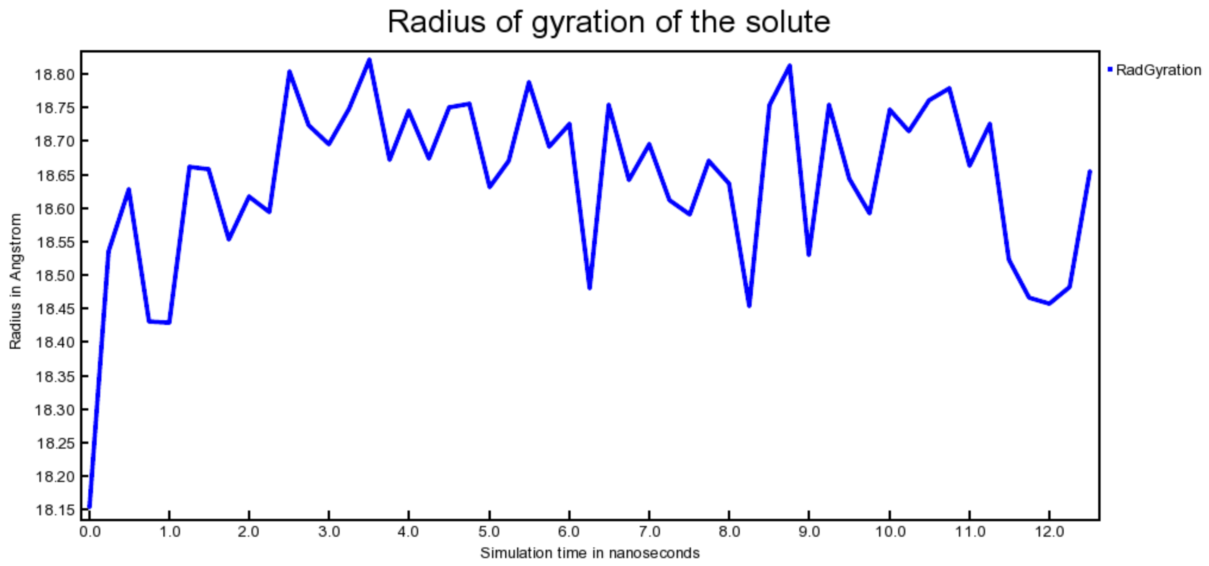

Figure S6. Radius of gyration of the solute [vertical axis] as a function of simulation time [horizontal axis]

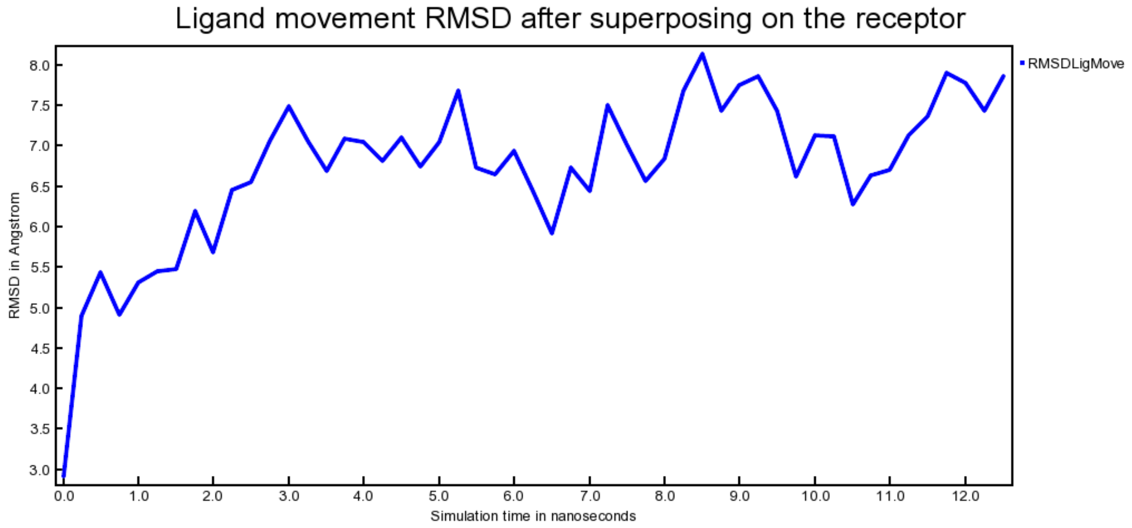

Figure S7. Ligand movement root mean square deviation (RMSD) after superposing on the receptor [vertical axis] as a function of simulation time [horizontal axis].
